# Collagen-chitosan-hydroxyapatite composite scaffolds for bone repair in ovariectomized rats

**DOI:** 10.1038/s41598-022-24424-x

**Published:** 2023-01-02

**Authors:** Erivelto Luís Chacon, Mirella Romanelli Vicente Bertolo, Ana Maria de Guzzi Plepis, Virginia da Conceição Amaro Martins, Geovane Ribeiro dos Santos, Clovis Antônio Lopes Pinto, André Antônio Pelegrine, Marcelo Lucchesi Teixeira, Daniela Vieira Buchaim, Fabricio Montenegro Nazari, Rogerio Leone Buchaim, Gustavo Tenório Sugano, Marcelo Rodrigues da Cunha

**Affiliations:** 1 Department of Morphology and Pathology, Jundiai Medical School, Jundiai, 13202-550 Brazil; 2 Sao Carlos Institute of Chemistry, University of Sao Paulo (USP), Sao Carlos, 13566-590 Brazil; 3 Interunits Graduate Program in Bioengineering (EESC/FMRP/IQSC), University of Sao Paulo (USP), Sao Carlos, 13566-970 Brazil; 4 Department of Implant Dentistry, Faculdade Sao Leopoldo Mandic, Campinas, 13045-755 Brazil; 5 Prosthodontics Department, Faculdade Sao Leopoldo Mandic, Campinas, 13045-755 Brazil; 6 Postgraduate Program in Structural and Functional Interactions in Rehabilitation, Postgraduate Department, University of Marilia (UNIMAR), Marilia, 17525-902 Brazil; 7 Teaching and Research Coordination of the Medical School, University Center of Adamantina (UNIFAI), Adamantina, 17800-000 Brazil; 8 Department of Biological Sciences, Bauru School of Dentistry (FOB), University of Sao Paulo (USP), Bauru, 17012-901 Brazil; 9 Graduate Program in Anatomy of Domestic and Wild Animals, Faculty of Veterinary Medicine and Animal Science (FMVZ), University of Sao Paulo (USP), Sao Paulo, 05508-270 Brazil

**Keywords:** Musculoskeletal system, Periodontitis, Preclinical research, Bone

## Abstract

Lesions with bone loss may require autologous grafts, which are considered the gold standard; however, natural or synthetic biomaterials are alternatives that can be used in clinical situations that require support for bone neoformation. Collagen and hydroxyapatite have been used for bone repair based on the concept of biomimetics, which can be combined with chitosan, forming a scaffold for cell adhesion and growth. However, osteoporosis caused by gonadal hormone deficiency can thus compromise the expected results of the osseointegration of scaffolds. The aim of this study was to investigate the osteoregenerative capacity of collagen (Co)/chitosan (Ch)/hydroxyapatite (Ha) scaffolds in rats with hormone deficiency caused by experimental bilateral ovariectomy. Forty-two rats were divided into non-ovariectomized (NO) and ovariectomized (O) groups, divided into three subgroups: control (empty defect) and two subgroups receiving collagen/chitosan/hydroxyapatite scaffolds prepared using different methods of hydroxyapatite incorporation, in situ (CoChHa1) and ex situ (CoChHa2). The defect areas were submitted to macroscopic, radiological, and histomorphometric analysis. No inflammatory processes were found in the tibial defect area that would indicate immune rejection of the scaffolds, thus confirming the biocompatibility of the biomaterials. Bone formation starting from the margins of the bone defect were observed in all rats, with a greater volume in the NO groups, particularly the group receiving CoChHa2. Less bone formation was found in the O subgroups when compared to the NO. In conclusion, collagen/chitosan/hydroxyapatite scaffolds stimulate bone growth in vivo but abnormal conditions of bone fragility caused by gonadal hormone deficiency may have delayed the bone repair process.

## Introduction

Natural fracture healing is a complex process that involves the formation, growth, and remodeling of bone for correct repair^1^. However, autologous grafts are indicated for injuries accompanied by marked loss of bone mass, which are considered the gold standard^2,3^. Natural or synthetic biomaterials can also be used as alternatives in clinical situations that require a support for cell growth^4^. Tissue engineering has been developing scaffolds that enable cell adhesion and proliferation^5,6^. Among these scaffolds, there are polymers that, due to their three-dimensional structures and characteristics similar to biological tissues, have the ability to allow cell growth^7^.

In vitro studies using collagen scaffolds have confirmed the suitability of polymers for bone reconstruction^8,9^, as well as for the regeneration of bone defects created experimentally in rats^10,11^. The advantages of collagen include properties such as biocompatibility, biodegradability, and cell interaction capacity^12^.

Collagen is the main structural protein found in the extracellular matrix of connective tissues and can be used in its native or reconstituted form^13^. Its properties can be modified by physicochemical or enzymatic processes, which result in a positively or negatively charged collagen matrix^14,15^.

Scaffolds based on collagen and hydroxyapatite have been developed for use in bone repair^6^ based on the concept of biomimetics^16^, improving their mechanical properties and biocompatibility^17^. The morphology of hydroxyapatite in the form of nanometric particles is similar to that of mineral crystals found in bone tissue, a feature that contributes to cell growth on scaffolds^18,19^. However, a homogenous distribution of the components and appropriate porosity are difficult to obtain, a fact that may compromise osteogenesis when hydroxyapatite particles are used for the treatment of skeletal defects. In this respect, collagen can be combined with other polymers to improve the composition of the scaffold and thus facilitate bone growth^20,21^, justifying the alternative use of nano-hydroxyapatite and chitosan as scaffolds in bone tissue engineering studies^6^.

The structure of chitosan is similar to that of glycosaminoglycans, glycoproteins, and glycolipids present in the extracellular matrix of biological tissues. Chitosan is a cationic biopolymer that is obtained by partial deacetylation of chitin, which is found in the structure of crustaceans, insects and mollusks^22^ and in the cell wall of fungi^23–26^. Chitosan has several clinical applications such as drug delivery, surgical threads and wound healing materials due to its hemostatic properties ^6,27^. In addition, chitosan exhibits the essential characteristics for a scaffold material since it is nontoxic and exerts antimicrobial activity^28–30^. In view of its properties and three-dimensional structure, this natural polymer has been used in experimental studies of bone reconstruction, which demonstrated its biocompatibility and biodegradability^7,31^, as well as important osteoconductive properties^11,30^.

Despite the indications of collagen/chitosan/hydroxyapatite scaffolds for bone repair, some factors may compromise the expected results of these materials when used in situations of bone fragility since, according to Frayssinet (1992), the health conditions of bone tissue are essential for osseointegration of the implant^32^. For example, the bone architecture is compromised in osteoporosis^32^. Causes of this disease include smoking^33^, alcoholism^34^, sedentarism^35^ and hormonal disorders resulting from early menopause or late menarche^36^.

The risk of osteoporosis is also increased in the case of ovariectomy since female gonadal hormones such as estrogen participate in bone growth and mineralization^37,38^. Therefore, the aim of the present study was to evaluate the osteoregenerative capacity of collagen/chitosan/hydroxyapatite-based scaffolds, prepared by two different methods of hydroxyapatite incorporation, in order to determine the ideal material for grafting of defects in healthy bone and fragile bone due to gonadal hormone deficiency.

## Results and discussion

### Analysis of the scaffolds

**Figure 1:**
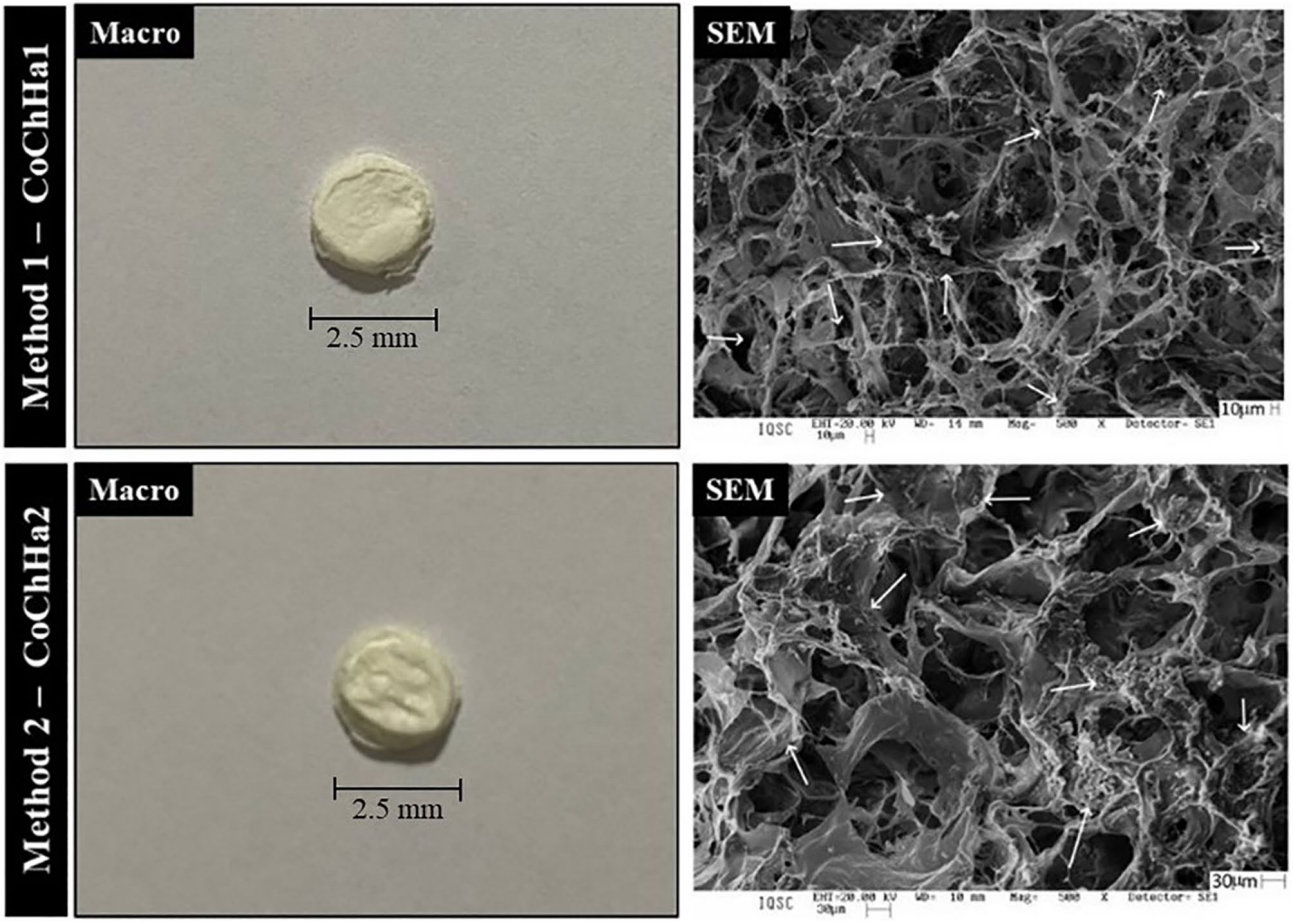
Macroscopic images of the scaffolds (left) and surface scanning electron photomicrographs (SEM, right) at ×500 magnification. Hydroxyapatite (arrows) was distributed throughout the polymer matrix.

Figure 1 shows the scaffolds and their respective morphologies obtained with the different methods of hydroxyapatite incorporation. The macro images showed no visual differences between the two scaffolds, both presented as three-dimensional structures of white color, similar size, and porous and homogeneous aspect. The porous structures observed in SEM images showed that hydroxyapatite (arrows) was present and distributed throughout the polymeric matrix in both cases, first indication that both adopted hydroxyapatite incorporation methods were successful.

Bertolo et al. (2019) found that, although not significant, CoChHa1 scaffolds had pores with smaller diameters (17.7 ± 6.8 µm) than CoChHa2 scaffolds (21.1 ± 8.5 µm)^6^. Moreover, the distribution of these pores was more homogeneous for CoChHa2 (around 33.33% of the pores with 20–30 µm of diameter), indicating that the method of phosphate incorporation into the scaffolds had a great influence on their morphological properties, which may affect the regenerative behavior of the scaffolds in vivo^6^. The pores of the scaffolds used in this study were significantly smaller than those found by Rahman et al. (2019), who prepared scaffolds of rabbit collagen, shrimp chitosan and bovine hydroxyapatite for restoration of defected maxillofacial mandible bone and found pores ranging from 101.69 ± 17 µm to 273.43 ± 49 µm^39^.

### Macroscopic and radiologic analysis of the bone defect

**Figure 2:**
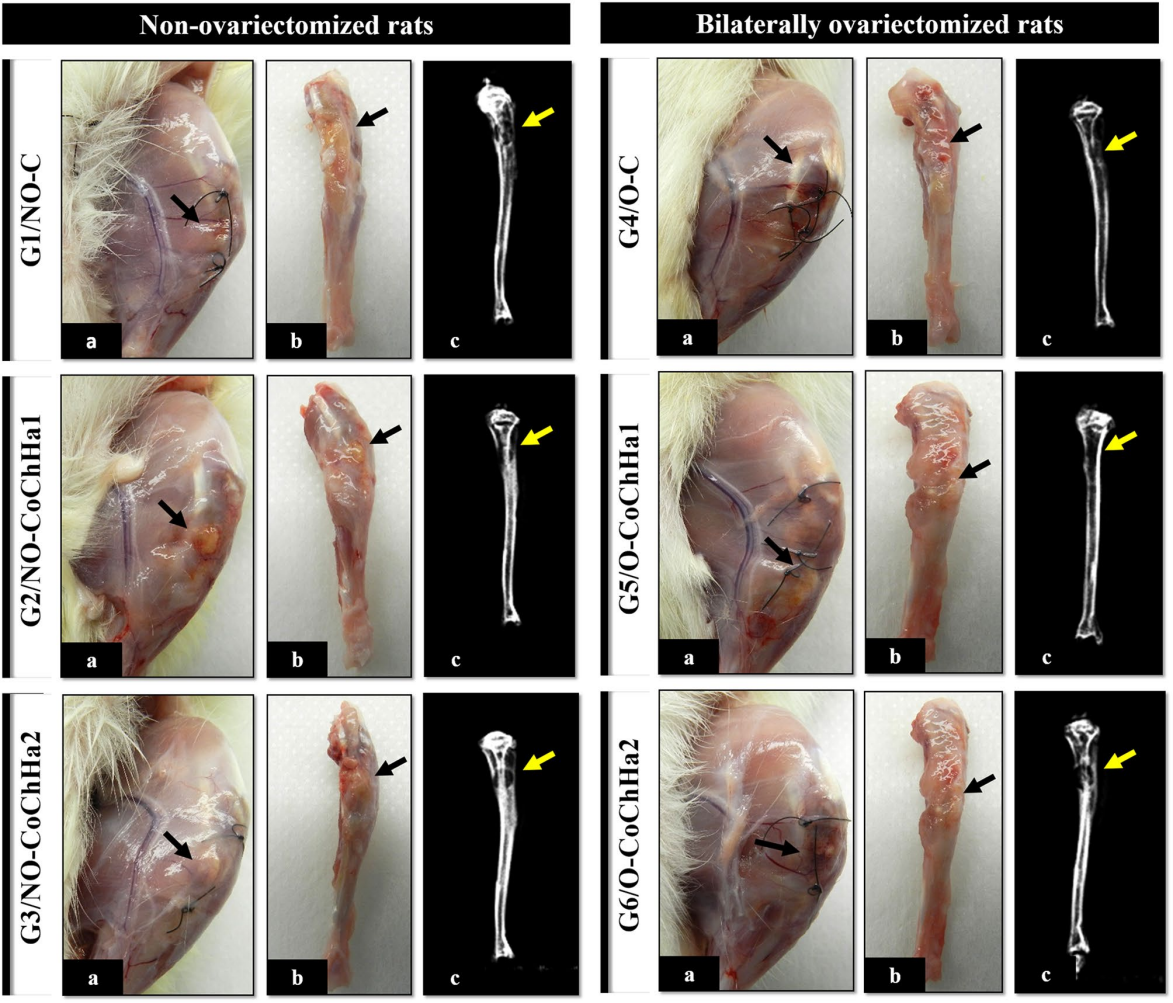
Macroscopic and radiologic images of the groups of non-ovariectomized and bilaterally ovariectomized rats. Note the absence of infections and bone rarefaction that would indicate an intense immune response to the scaffolds. (**a**) Macroscopic image of the defect area in the left tibia after induced painless death. (**b**) Macroscopic image of the dissected left tibia. (**c**) Radiographic image of the left tibia. The arrows indicate the bone defect area in the proximal metaphysis of the left tibia.

Macroscopic analysis of the defect area in all rats showed good healing of the soft tissues, as demonstrated by the absence of necrosis, cysts, fibromatosis, abscesses and any evidence of a subcutaneous or deeper tissue inflammatory process. In the bone area, there were also no signs of osteomyelitis, secondary fractures or pseudarthrosis that would suggest any infectious complications (Fig. 2). These good outcomes of the animals may be explained by the fact that the experimental protocol followed the ARRIVE guidelines and principles of the NC3Rs initiative. The animals were monitored for the expression of pain by observing whether the animal was apathetic, depressed, aggressive, or overexcited, such traits being variable in their usual behavior. Possible changes in gait, posture or facial expression were also observed, and appearance, water intake, feeding and clinical symptoms were investigated. There were no complications that needed to be reported and no diseases or signs recommending the removal of an animal (clinical outcome) were observed^40^. In addition, our research group has experience in the method used, as demonstrated by previously published studies^11,41–43^.

The radiographic images showed a round radiolucency of the defect area in the proximal tibial metaphysis and radiopacity of the cortical margins of the tibia, demonstrating the absence of deformities or any other type of change in the bone structure (Fig. 2). These results are compatible with studies on the tibial metaphysis, an experimental model widely used for the investigation of bone repair and biomaterial filling, especially in non-critical defects^44,45^. Thus, the two types of scaffolds used in this study showed biocompatibility in critical bone defects, confirming the biocompatibility of the materials used, both polymers and the inorganic phase incorporated on them^19^. Zugravu et al. (2012) also reported this advantage for collagen/chitosan/hydroxyapatite scaffolds in in vitro studies^46^. Furthermore, the findings demonstrated that gonadal hormone deficiency did not exacerbate the local inflammatory response.

### Morphological analysis of the bone defect area

**Figure 3:**
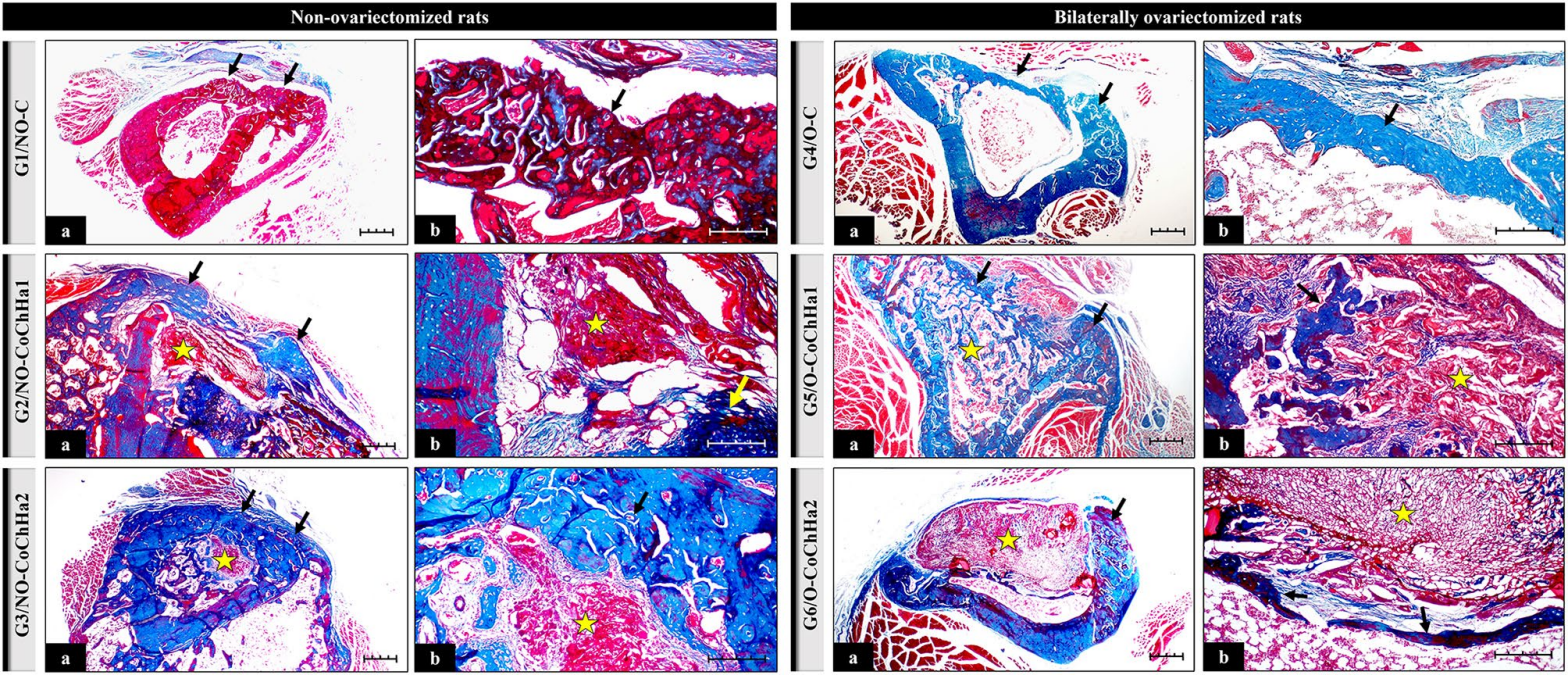
Photomicrographs of histological slides stained with Masson’s trichrome at ×4 (**a**) and ×10 magnification (**b**) obtained from the groups of non-ovariectomized and bilaterally ovariectomized rats. The arrows point to areas of bone neoformation. The star indicates the presence of biomaterial undergoing bioresorption. Scale bar: 20 µm.

The formation of new bone in the defect experimentally induced in the proximal tibial metaphysis of rats was observed in all groups analyzed; however, its morphology differed between the control groups and the experimental groups treated with the biomaterial and submitted to ovariectomy. The new bone projected from the margins of the defect but exhibited peculiar histological characteristics that differed between the groups studied and from the original bone of each rat. New formed bone contained lacunae filled with osteocytes that were arranged in various directions. The medullary canal was preserved and filled with hematopoietic tissue and bone trabeculae. Resorption of the biomaterials differed between the grafted groups (Fig. 3).

There is still a preference for the use of autologous grafts for bone grafting; however, due to the need for two surgical beds (donor and recipient area), morbidity and limited availability, advantages arise in the use of synthetic biomaterials for bone tissue regeneration^47^. Three-dimensional scaffolds are particularly interesting in tissue engineering since they can act as structures to accommodate cells and support tissue growth, providing support for cell adhesion, proliferation, and migration^48^. The creation of a bone defect triggers a sequence of events in the local microenvironment, including the migration of inflammatory and proliferative cells of bone tissue, compatible with the remodeling process. These events allow the bone to respond and to adapt to functional changes, as observed in the present study^49^. The resorption rate of biomaterials differs depending on the material used. For example, particulate dentin grafts are characterized by a high resorption rate after 24 months as well as by bone substitution without inflammation. Since dentin particles have open tubes, capillaries can access their interior, resulting in rapid resorption^50^.

There is currently increasing interest in composites consisting of natural polymers (like collagen and chitosan) and hydroxyapatite, which form biocompatible scaffolds with interconnected pores that have a satisfactory osteogenic potential^39,51,52^. Collagen type I is the main component of the extracellular matrix and bone tissue in humans, while hydroxyapatite is the second most abundant component in bone^39^, which can be prepared synthetically with specific nano-sized pores for adequate deposition in the 3D microarchitecture of the scaffolds, called nano-calcium phosphate^6^.

Collagen matrices containing nano-calcium phosphate have shown osteogenic potential in critical bone defects^53^. Recently, synthetic calcium phosphate has been reported to stimulate biomineralization in collagen-based bone substitutes^54^. There is also evidence that chitosan plays an important role in the strengthening of the microarchitecture of these polymer matrices, whose biodegradability can be adapted according to the proportion of chitosan or hydroxyapatite^52,55^.

Specifically, in non-ovariectomized rats (G1, G2 and G3), the formation of irregular and immature bone was observed in the control group (G1, NO-C), including joining of the margins of the bone defect that contained cavities and spaces but without the interposition of connective tissue. In G2 (NO-CoChHa1), remnants of the biomaterial surrounded almost entirely by newly formed bone were found; however, bone neoformation was not sufficient to fill the entire defect due to the presence of connective tissue in the defect area. In G3 (NO-CoChHa2), the defect closed due to the volume of new bone formed, in a more compact way and promoting the union of the lesion margins, without the presence of connective tissue. Also in G3, there were remnants of the biomaterial inside the medullary canal, which, in turn, was filled with hematopoietic tissue, and several bone trabeculae. This feature was only observed in this group (Fig. 3). These microscopic findings agree with studies on bone repair that report the gradual centripetal substitution of the implanted biomaterial with newly formed bone, demonstrating the biocompatibility of the scaffolds used in this experiment^56–58^.

Histological analysis indicated a superior osteogenic potential of CoChHa2, a scaffold in which calcium phosphate was incorporated ex situ into the mixture of collagen gel and chitosan powder. The SEM images of this scaffold had already revealed a porous morphology suitable for growth and cell proliferation. The more homogeneous pore distribution might have been a factor to improve the osteogenic potential of CoChHa2, which was also about 7% less porous and absorbed around 500% less phosphate buffer saline (PBS) than CoChHa1 scaffolds. Moreover, X-rays diffraction patterns of hydroxyapatite (2θ = 32°) were present in both diffractograms in the study of Bertolo et al. (2019)^6^; however, hydroxyapatite influence on bone formation might have been greater in CoChHa2, that presented more intense and better-defined peaks (i.e., greater crystallinity, less influenced by collagen and chitosan presence).

According to the histological results, CoChHa2 stimulated greater bone neoformation in the tibial defect area of the animals. Regarding CoChHa1, bone neoformation also occurred around the defect area, but to a lesser extent. In conclusion, the two scaffolds studied here can be indicated for bone repair, since they both present interconnected pores and three-dimensional structures with proven presence of hydroxyapatite; however, there are differences in the time and rate of bone formation, factors directly related to the morphological and physicochemical properties (porosity, absorption, degradation) of the materials, as well as with the availability of the calcium phosphate phase. Scaffolds with more homogeneous porous and with hydroxyapatite more available in the polymeric matrix tend to accelerate the osteogenic process, which is a good feature when working with short recovery periods.

In ovariectomized rats, the formation of thinner bone along the defect was observed in G4 (O-C); however, the space was not completely closed given the presence of connective tissue. In G5 (O-CoChHa1), the young bone was compact at the margins of the defect but more trabecular and bordering the lower surface of the biomaterial which, in turn, was not completely reabsorbed. In G6 (O-CoChHa2), the biomaterial remained intact, with little bone formation around it, and a predominance of connective tissue was thus observed (Fig. 3). Analysis of the scaffolds in ovariectomized rats treated with CoChHa1 (G5) and CoChHa2 (G6) showed less bone formation in the two groups when compared to the respective non-ovariectomized groups; nevertheless, both scaffolds exerted a satisfactory effect as demonstrated by the onset of bone repair, although the process was slower. Evaluating polymer scaffolds in the femur of ovariectomized rats, Cunha et al. (2008) concluded that bone formation is dependent on the properties of the biomaterials as well as on the quality of the host bone tissue^59^.

Preclinical studies using an experimental model similar to that employed in this study have attempted to improve the formation of new bone in rats submitted to ovariectomy (or other models of osteoporosis induction) using tissue engineering techniques. Zhang et al. (2022) developed a new class of copper-alloyed titanium (TiCu) alloys with excellent mechanical properties and biofunctionalization^60^. The authors induced osteoporosis in rats to study the effect of osseointegration and the underlying mechanism of TiCu. The alloy increased fixation stability, accelerated osseointegration, and thus reduced the risk of aseptic loosening during long-term implantation in the osteoporosis environment. The study of Zhang et al. (2022) was the first to report the role and mechanism of a copper-alloyed metal in promoting osseointegration in the osteoporosis environment^60^.

Current technologies permit to connect different active functional groups by modifying their configuration or surface. These changes can significantly broaden the range of applications and efficacy of chitosan polymers^61^. Chitosan, calcium phosphate and collagen and their combination in composite materials meet the required properties (biocompatible, bioactive, biodegradable, and multifunctional) and can promote biostimulation for tissue regeneration^52^. In some situations of alteration by pathologies, the repair process and healing rate may be compromised even when these biomaterial are used^62,63^.

**Figure 4:**
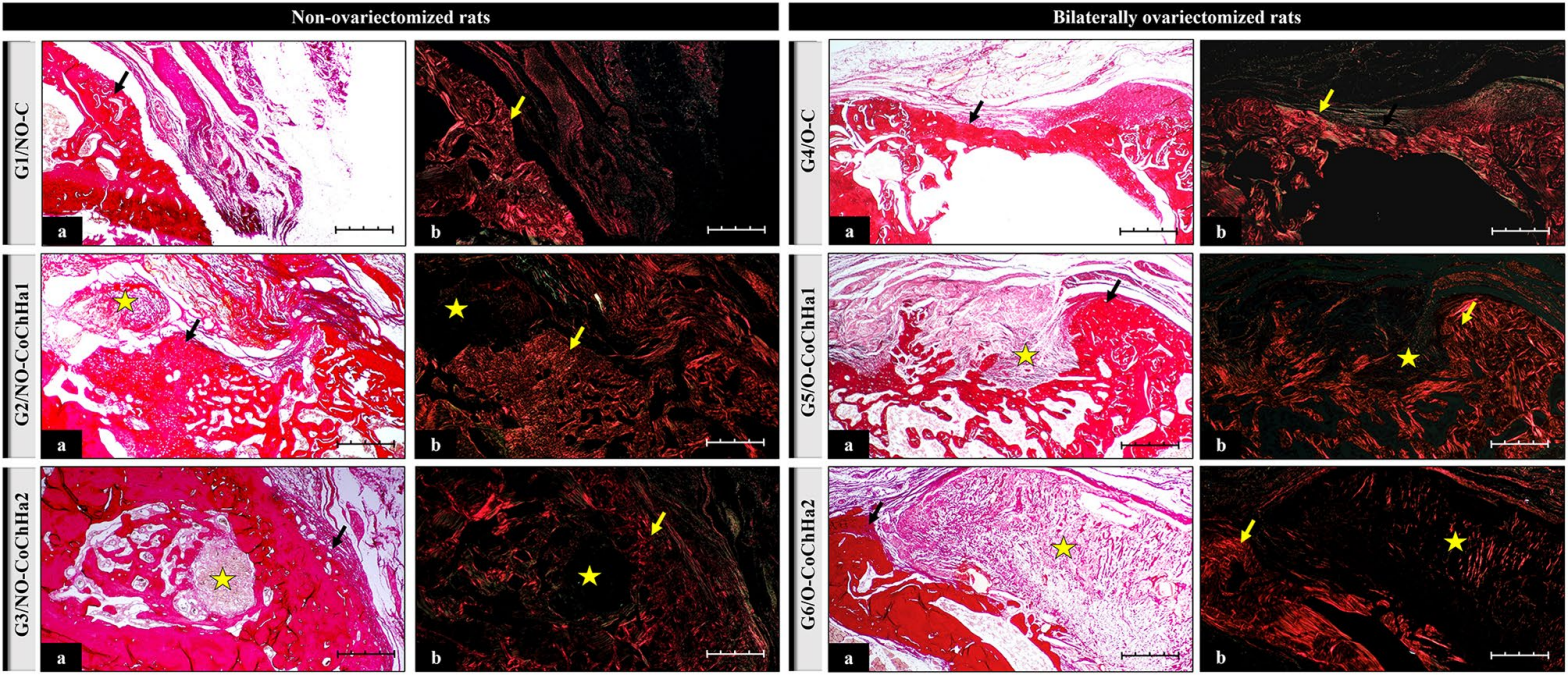
Photomicrographs of histological slides stained with Picrosirius red obtained from the groups of non-ovariectomized and bilaterally ovariectomized rats and examined under an optical microscope (**a**) and under polarized light (**b**) at ×10 magnification. The black arrows point to areas of bone neoformation. The yellow arrows indicate the presence of collagen fibers in the extracellular matrix. The star indicates the area occupied by remnant biomaterial after bioresorption. Scale bar: 20 µm.

Staining with Picrosirius red under polarized light showed collagen birefringence in the extracellular matrix of tissue present in the defect area of all groups (Fig. 4). Picrosirius red is a dye that selectively stains connective tissue, enabling the qualitative analysis of collagen fibers. When observed under polarized light, this stain permits to differentiate especially type I and type III fibers based on the difference in interference colors, intensity and birefringence of the stained tissues^64^. New bone formation was characterized by red–orange birefringence, corresponding to the osteoid matrix, which gradually changed to yellow-green. Similar findings have been reported by Della Colletta et al. (2021)^65^.

### Histomorphometric and statistical analysis of newly formed bone volume in the defect area

The following means and standard deviations of the percent volume of newly formed bone in the tibial defect were obtained for the groups studied: 69.20 ± 3.3 (G1); 67.08 ± 4.5 (G2); 92.12 ± 0.5 (G3); 44.63 ± 5.3 (G4); 43.42 ± 9.9 (G5), and 44.59 ± 5.2 (G6). Statistical analysis revealed no significant difference between the ovariectomized groups (G4 vs. G5, G4 vs. G6, G5 vs. G6), while comparison of the non-ovariectomized groups showed a higher percent volume in G3 (NO-CoChHa2). Comparison of non-ovariectomized animals with their respective ovariectomized counterparts (G1 vs. G4, G2 vs. G5, G3 vs. G6) revealed a higher bone volume in the non-ovariectomized groups.

These results demonstrate that the biomaterials were unable to contribute to bone volume gain when a hormonal component resulting from experimental ovariectomy is involved. On the other hand, the bone volume increase was significant in non-ovariectomized animals grafted with the CoChHa2 scaffold (collagen/chitosan/hydroxyapatite, method 2). The success of biomaterials for fracture fixation in osteoporotic patients, or simply for bone augmentation, is compromised by poor bone quality and decreased osteoblastic activity. Further research, driven by clinical demand, is therefore needed to address this issue. The growing elderly population and the different associated pathologies require studies that involve close cooperation between basic research, applied research, clinical research, and regulatory bodies^66^.

## Conclusions

This in vivo study aimed to evaluate the osteoregenerative capacity of collagen/chitosan/hydroxyapatite scaffolds, obtained by two different methods of hydroxyapatite preparation, in defects created in healthy tibial bone of rats and in fragile bone due to gonadal hormone deficiency (ovariectomy). Macroscopic, radiologic, histomorphological, and morphometric assessment showed that the new bone projected from the margins of the defect in a centripetal manner in all animals. In ovariectomized rats, the new bone forming along the defect was thinner and did not completely close the surgical cavity, as indicated by the presence of connective tissue 5 weeks after surgery.

In non-ovariectomized animals, there was a significant increase in new bone in G3 that received the CoChHa2 scaffold. Thus, this type of scaffold was the best option for in vivo bone growth; however, CoChHa1 can also be used for bone repair, with differences in the time and rate of bone formation. The abnormal conditions of bone fragility caused by gonadal deficiency can delay the process of bone repair, even when scaffolds are used in the search for cell growth and proliferation.

## Materials and methods

Collagen was extracted from bovine tendon purchased at Casa de Carnes Santa Paula, Sao Carlos, SP. For chitosan preparation, squid pens (*Doryteuthis* spp.) were used as a source of β-chitin (obtained at Miami Comércio e Exportação de Pescados Ltda, Cananéia, SP, Brazil). All solvents and reagents used for the preparation of the scaffolds were of analytical grade.

### Collagen obtention

Collagen obtention started with the treatment of the bovine tendon in an alkaline solution (with chlorides and sulfates of Na^+^, K^+^, and Ca^2+^) for 72 h at 25 °C^67^. The excess salts were thenremoved by washes in boric acid (H_3_BO_3_) and EDTA solutions and in deionized water. Finally, collagen was extracted in an acetic acid (HAc) solution (pH 3.5). Its concentration of 0.98 ± 0.23% was determined by lyophilization.

### Chitosan obtention

The squid pens (*Doryteuthis* spp.) used as a source of β-chitin were submitted to the method of deproteinization and deacetylation in order to obtain chitosan^67^. The reaction yield was 27.8% and the chitosan acetylation degree and molecular mass were 6.7% and 327 kDa, respectively, as previously described and characterized by Bertolo et al. (2019)^6^. For the subsequent scaffold preparation, chitosan was solubilized in 1% (w/w) HAc for 24 h, leading to a 1% (w/w) chitosan gel^6^.

### Preparation and characterization of collagen/chitosan/hydroxyapatite scaffolds

Two different methods were adopted to prepare the collagen/chitosan/hydroxyapatite scaffolds, according to the methodology described by Bertolo et al. (2019)^6^. The main difference between the methods concerns the in situ (i.e., in the polymeric matrix) or ex situ preparation of calcium phosphate, as described in detail below. In both methods, the collagen: chitosan ratio was kept at 1:1 and the amount of calcium phosphate was 35% of the dry polymer mass in the scaffolds.

### CoChHa1–collagen/chitosan/hydroxyapatite, method 1

For calcium phosphate formation, CaCl_2_ and (NH_4_)_2_HPO_4_salts (0.2 mol L^−1^ and 0.12 mol L^−1^, respectively) were added to the 1% (w/w) chitosan gel. After 24 h, the pH of the gel was raised to 9.0 with 1.0 mol L^−1^ NH_4_OH. After 48 h of stirring at room temperature, excess salts were removed with water and the chitosan solution containing the calcium phosphate synthesized in situ was solubilized in HAc, pH 3.5. Finally, the solution was added to the 1% (w/w) collagen gel and homogeneous mixtures were obtained after 48 h under stirring. After removing the air, the mixtures were placed in Teflon^®^ molds, frozen, and lyophilized to obtain the scaffolds. The scaffolds were washed in phosphate-buffered saline (PBS), pH 7.4, and in deionized water, frozen, and lyophilized.

### CoChHa2–collagen/chitosan/hydroxyapatite, method 2

In this method, calcium phosphate was synthesized ex situ with CaCl_2_.2H_2_O and (NH_4_)_2_HPO_4_salts (0.05 mol L^−1^ and 0.03 mol L^−1^, respectively) in a 0.15% pectin solution. After calcination to eliminate the pectin matrix, the synthesized calcium phosphate (grain size of 21.4 nm) was added to the 1% (w/w) collagen gel and the mixture was kept under stirring for 60 min. Next, chitosan powder was added to the mixture, which was kept under stirring for 24 h. After removing the air, the mixtures were placed in Teflon^®^ molds, frozen, and lyophilized to obtain the scaffolds. The scaffolds were washed in PBS, pH 7.4, and in deionized water, frozen, and lyophilized.

### Scanning electron microscopy (SEM)

CoChHa1 and CoChHa2 morphology was observed with a Zeiss LEO 440 (Cambridge, England) equipment, with an Oxford detector (model 7060) and an electron beam of 20 kV. Prior the analysis, both scaffolds were placed in stubs with conductive carbon tape and covered with a gold layer (6 mm).

### Experimental design

Forty-two female Wistar rats (*Rattus norvegicus*), 12 weeks old and weighing on average 350 g, maintained at the animal bioterium of the Jundiai Medical School (FMJ/Brazil) were used in this study. The Ethics Committee on Animal Experimentation of FMJ approved the project (Protocol 276/2017).

**Figure 5:**
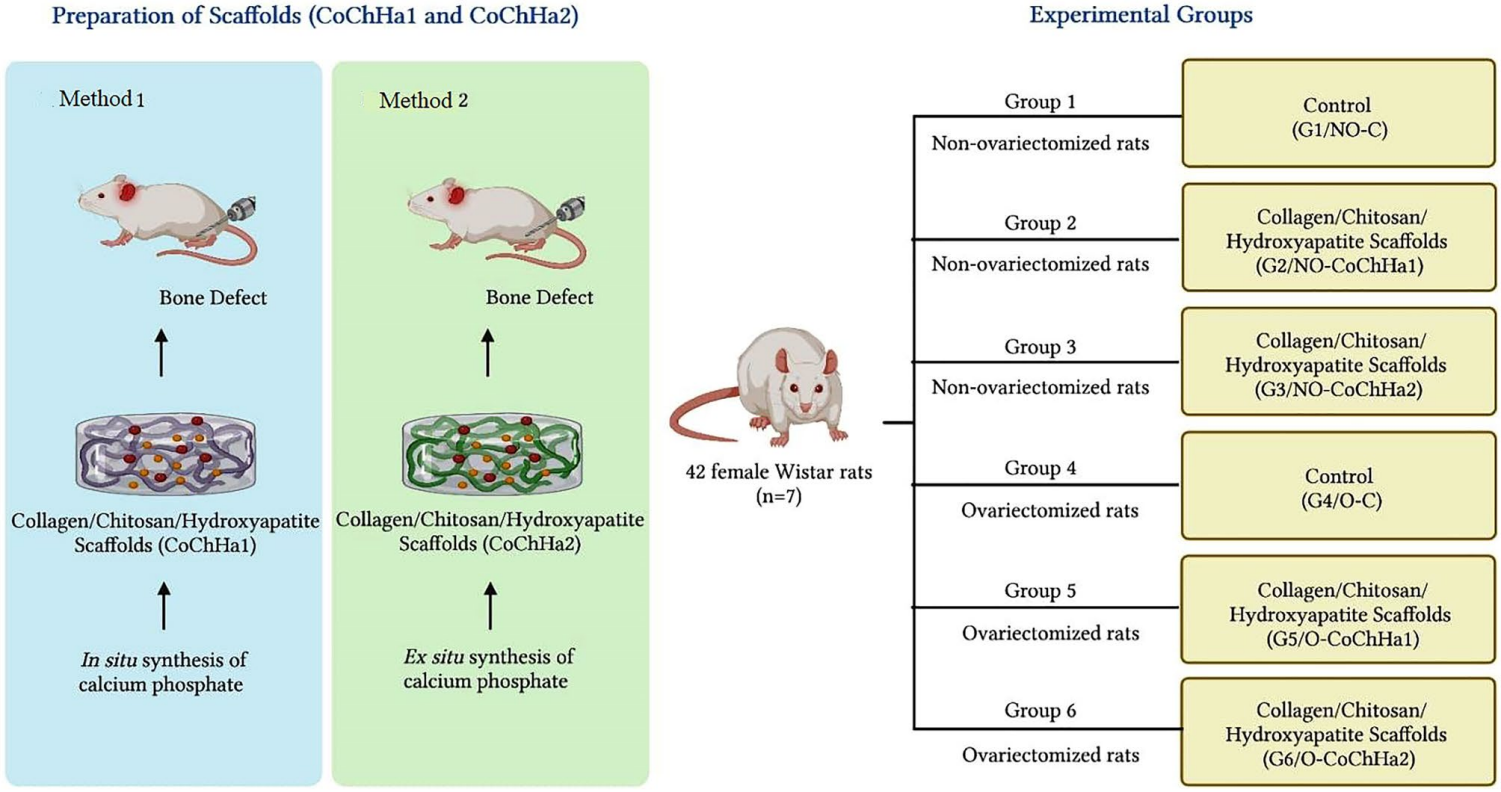
Schematic representation of the experimental design for formation of the experimental groups.

The rats were divided into healthy, non-ovariectomized groups (NO; G1, G2, G3) and bilaterally ovariectomized groups (O; G4, G5, G6), and then submitted to the surgical procedure for creation of a proximal metaphyseal defect in the left tibia. The animals were subdivided into control and experimental groups. In the latter groups, the tibial defect was grafted with two types of collagen/chitosan/hydroxyapatite scaffolds: Group 1 (G1, NO-control): non-ovariectomized rats without graft in the bone defect; Group 2 (G2, NO-CoChHa1): non-ovariectomized rats grafted with the CoChHa1 scaffold; Group 3 (G3, NO-CoChHa2): non-ovariectomized rats grafted with the CoChHa2 scaffold; Group 4 (G4, O-control): ovariectomized rats without graft in the bone defect; Group 5 (G5, O-CoChHa1): ovariectomized rats grafted with the CoChHa1 scaffold; Group 6 (G6, O-CoChHa2): ovariectomized rats grafted with the CoChHa2 scaffold (Fig. 5).

### Bilateral ovariectomy

**Figure 6:**
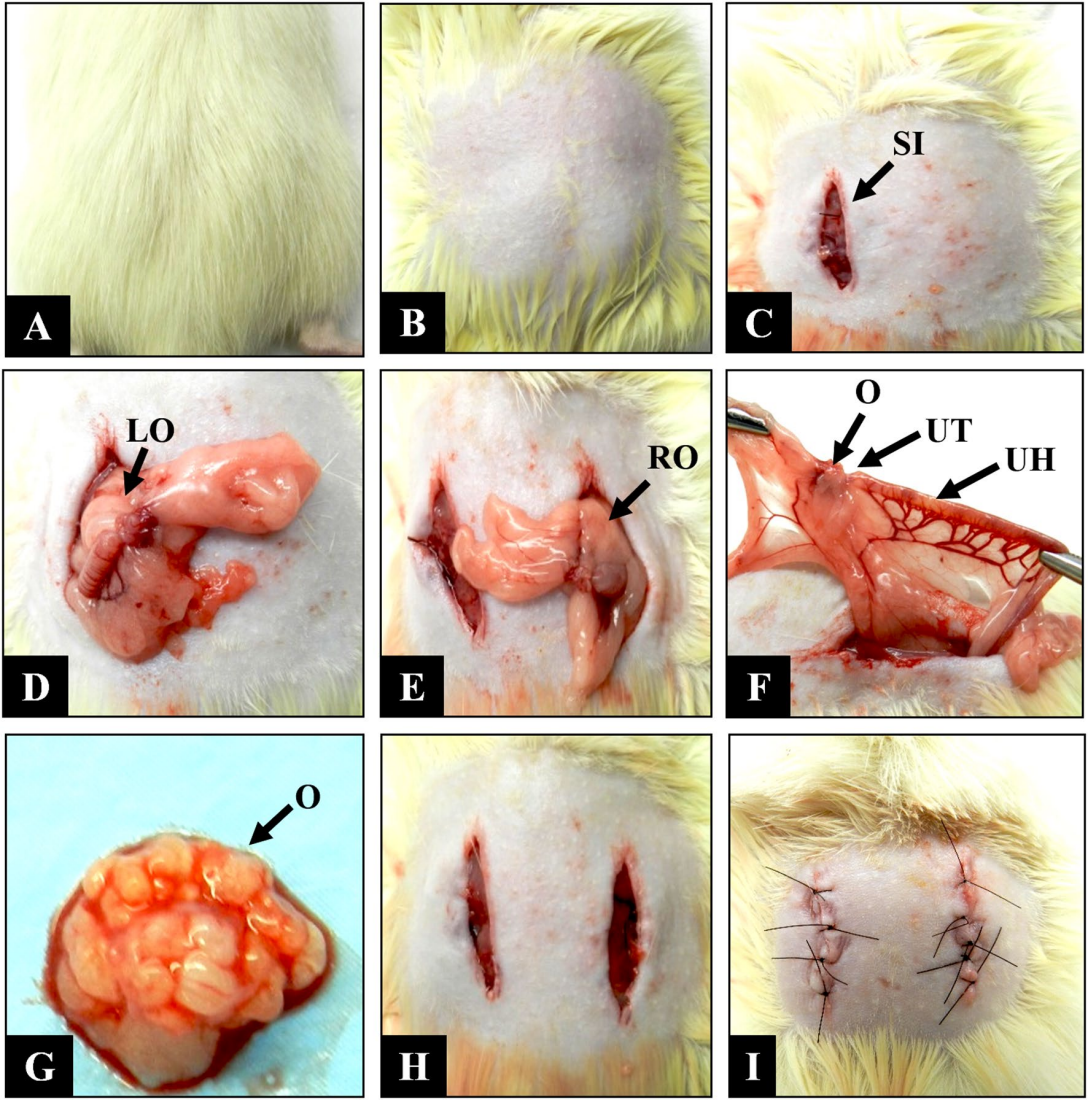
Ovariectomy of female Wistar rats. (**A**) Positioning of the animal in ventral decubitus. (**B**) Shaving of the lumbar region. (**C**) Longitudinal 10-mm surgical incision on the left side (SI). (**D**) Exploration of the left ovary (LO). (**E**) Contralateral posterior incision and exploration of the right ovary (RO). (**F**) Identification of the ovary (**O**), uterine tube (UT), and uterine horn (UH). (**G**) Dissection of the ovary (O). (**H**) Repositioning of the anatomical structures. (**I**) Suture of muscles and skin, respectively.

Rats of G4 and G6 were anesthetized by intramuscular injection (gluteus) of 0.1 ml/100 g body weight of xylazine (Vetaset^®^) and ketamine (Dopalen^®^) at a ratio of 1:1^10^. After the confirmation of anesthesia, the animals were placed in ventral decubitus for shaving and asepsis of the lumbar region with 2% chlorhexidine digluconate. A bilateral skin incision was made in the lumbar spine, elevating the skin and muscles in order to expose the retroperitoneum for removal of the ovaries. After removal, the soft tissues and skin were sutured (Ethicon^®^, Johnson & Johnson, São José dos Campos, SP, Brazil). In ovariectomized rats, surgery for creation of the proximal metaphyseal defect in the left tibia was performed 5 months after removal of the ovaries. This interval is sufficient to cause bone mineral loss due to estrogen deficiency^59,68^ (Fig. 6).

### Surgical technique for creation of a proximal metaphyseal defect in the left tibia

**Figure 7:**
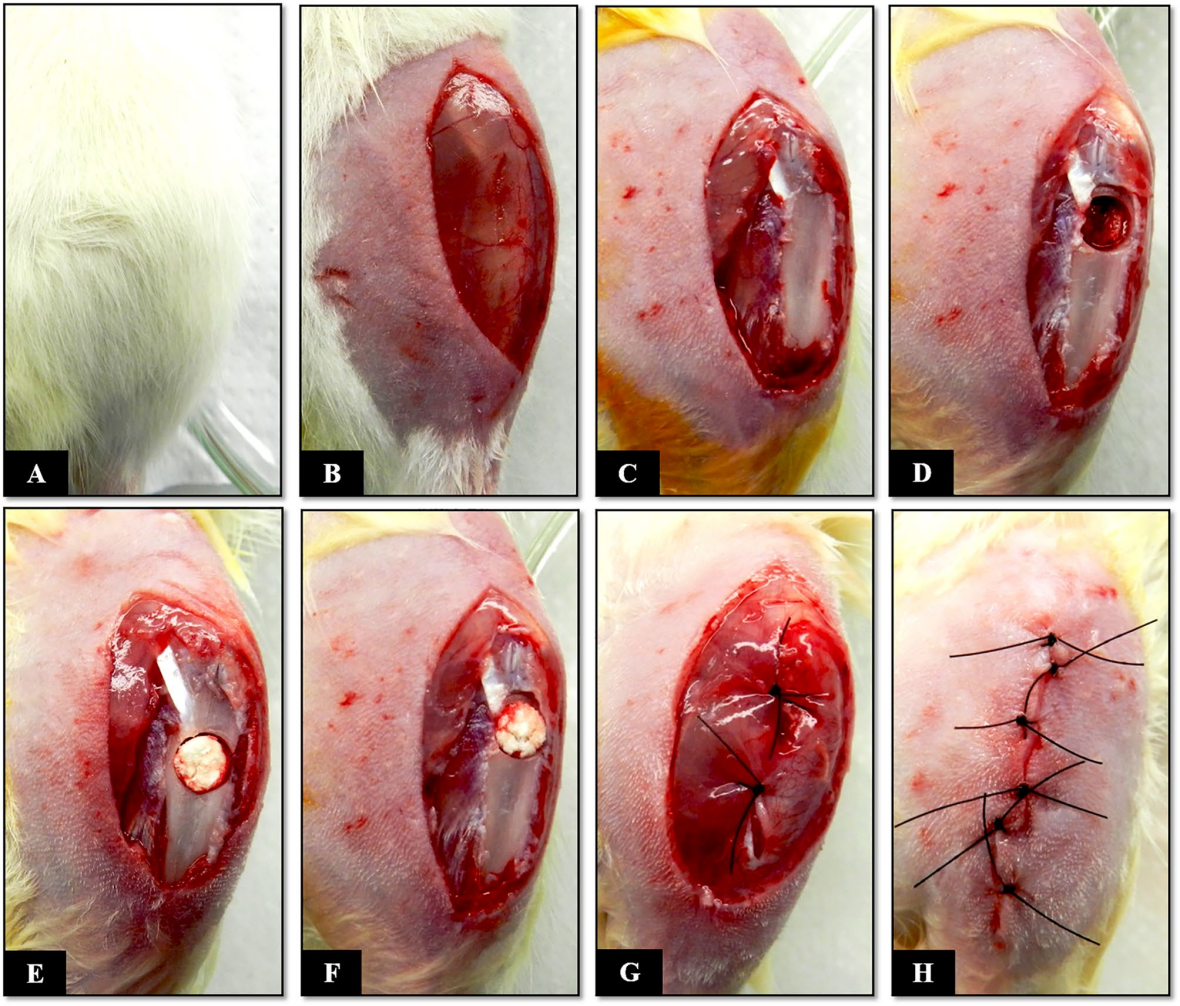
Surgical procedure for creation of a proximal metaphyseal bone defect in the tibia of Wistar rats. (**A**) Positioning of the rat in dorsal decubitus, with the left knee flexed. (**B**) Shaving of the left leg, followed by a longitudinal 15-mm skin incision. (**C**) Longitudinal incision in the muscle and periosteum. (**D**) Creation of a 2.5-mm bone defect in the proximal metaphysis of the left tibia. (**E** and **F**) Grafting with the CoChHa1 and CoChHa2 scaffolds. (**G**) Repositioning of the periosteum and muscular suture. (**H**) Skin suture.

Rats of all groups (G1 to G6) were anesthetized following the same protocol as used for the ovariectomy procedure but adding a subcutaneous dorsal application of Tramadol (2% Cronidor, Brazil). The animals were placed in dorsal decubitus for shaving and asepsis of the left hind leg with 2% chlorhexidine digluconate. After this step, a longitudinal skin incision was made to expose and separate the proximal muscles of the left leg. Once the proximal metaphysis of the left tibia was exposed, a bone defect measuring 2.5 mm in diameter was created with a trephine drill coupled to the pen of a mini-motor (Beltec LB-100, Brazil) until the medullary canal was reached. During this procedure, the area was continuously irrigated with saline to prevent local heating. The contents of the medullary cavity were curetted and the surgical site was washed with saline in order to remove bone remnants resulting from the technical method, thus avoiding any type of osteogenic induction that could compromise the histological analysis of bone repair. Next, the biomaterials studied were implanted in G2, G3, G5, and G6, while the bone defect remained empty in the control groups (G1 and G4). After the surgical, the soft tissues including periosteum, muscles, fasciae, and skin were sutured (Fig. 7).

In both surgical procedures (ovariectomy and tibial defect creation), rifamycin spray (Rifotrat^®^, Brazil) was applied to the surgical site as antibiotic. Each animal then received an intramuscular dose of 0.1 mg/100 g body weight of antibiotic (Pentabiotic Veterinário Pequeno Porte, Fort Dodge^®^, Brazil) for one week after surgery. In addition, the rats were constantly monitored and received paracetamol diluted in water in the “feeder” during the postoperative period^69^.

### Macroscopic, radiologic and histomorphometric analysis of the defect area

Five weeks after surgery, the animals were sacrificed by an overdose of the intramuscular anesthetic, followed by pneumothorax. The left tibia was removed and photodocumented for analysis of the clinical conditions of the defect area. Next, the samples were radiographed to assess the integrity and radiodensity of the defect area.

The left tibiae were kept in formaldehyde solution and were then decalcified to be reduced in the defect areas. The samples were submitted to routine histological methods. Semi-serial 5-µm sections were stained with Masson’s trichrome for the characterization of bone neoformation and with Picrosirius red (saturated aqueous solution of picric acid plus 0.1 g Sirius red F3B, Bayer^®^, Germany) for the identification of fibrillar extracellular matrix components by polarized light microscopy.

For quantification of the volume of newly formed bone in the defect area of the animals, the Motic Images Plus 2.0 ML software was used to delimit the total area of the bone defect and of newly formed bone. These values were used to obtain the percent volume of bone formed in the defect area. These data were entered into the BioEstat 5.3 software and ANOVA followed by the Tukey test was used for comparison between groups, adopting a level of significance of p < 0.05.

### Institutional review board statement

The study was conducted according to the guidelines of the Declaration of Helsinki, ARRIVE guidelines and approved by the Institutional Review Board (or Ethics Committee) of the Animal Research Ethics Committee of the Faculty of Medicine of Jundiaí, Brazil (CEUA/FMJ), protocol code CEUA/FMJ No. 276/2017.

## Data Availability

The data presented in this study are available on request from the corresponding author.
